# Aristolochic Acid-Induced Nephrotoxicity: Molecular Mechanisms and Potential Protective Approaches

**DOI:** 10.3390/ijms21031157

**Published:** 2020-02-10

**Authors:** Etienne Empweb Anger, Feng Yu, Ji Li

**Affiliations:** Department of Clinical Pharmacy, School of Basic Medical Sciences and Clinical Pharmacy, China Pharmaceutical University, Nanjing 211198, China; angetipharm9@gmail.com (E.E.A.); yufengcpu@163.com (F.Y.)

**Keywords:** aristolochic acid, aristolochic acid nephrotoxicity, DNA adducts formation, upper urothelial carcinoma, potential protective mechanisms

## Abstract

Aristolochic acid (AA) is a generic term that describes a group of structurally related compounds found in the Aristolochiaceae plants family. These plants have been used for decades to treat various diseases. However, the consumption of products derived from plants containing AA has been associated with the development of nephropathy and carcinoma, mainly the upper urothelial carcinoma (UUC). AA has been identified as the causative agent of these pathologies. Several studies on mechanisms of action of AA nephrotoxicity have been conducted, but the comprehensive mechanisms of AA-induced nephrotoxicity and carcinogenesis have not yet fully been elucidated, and therapeutic measures are therefore limited. This review aimed to summarize the molecular mechanisms underlying AA-induced nephrotoxicity with an emphasis on its enzymatic bioactivation, and to discuss some agents and their modes of action to reduce AA nephrotoxicity. By addressing these two aspects, including mechanisms of action of AA nephrotoxicity and protective approaches against the latter, and especially by covering the whole range of these protective agents, this review provides an overview on AA nephrotoxicity. It also reports new knowledge on mechanisms of AA-mediated nephrotoxicity recently published in the literature and provides suggestions for future studies.

## 1. Introduction

Remedies prepared from Aristolochiaceae plants containing aristolochic acid (AA) under various pharmaceutical formulations, including pills, powder, granules, pellets, infusions, and capsules [1,2], have been used for decades throughout the world to treat diverse diseases, including hepatitis, urinary tract infection, vaginitis, upper respiratory tract infection, eczema, dysmenorrhea, arthralgia, hypertension, bronchitis, pneumonia, heart failure, and edema [3,4,5]. However, plants or botanical-derived products containing AA were reported to be nephrotoxic, mutagenic, and carcinogenic to humans [3,6]. The first observation of unusual renal disease which consisted of rapidly progressive tubulointerstitial nephritis was made in Belgium in the 1990s among young female patients [7]. The etiology of this renal disease was quickly associated with consumption of body weight-loss pills containing a Chinese herb, Gang Fang Ji [5,8,9]. In this slimming regimen, *Stephania tetrandra* (Han Fang Ji) had been mistakenly substituted by *Aristolochia fangchi* (Gang Fang Ji) [4,10]. This pathology was initially known as Chinese herb nephropathy (CHN) because of the origin (China) of the slimming pills. After identifying AA as the causative agent of this pathology [11,12], CHN was then appropriately termed aristolochic acid nephropathy (AAN) [13,14]. A similar situation in some southeastern regions of Europe previously identified as Balkan endemic nephropathy (BEN), was later described to be the result of AA consumption too [15,16].

**Figure 1:**
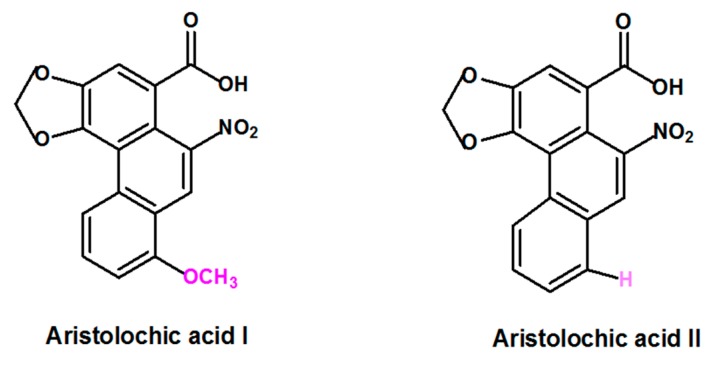
Formula of the most abundant and active of Aristolochic acids compounds: Aristolochic acid I and Aristolochic acid II.

Aristolochic acid (AA) is a generic term that refers to a structurally related group of nitrophenanthrene carboxylic acids derived from plants of the Aristolochiaceae family that includes several species. Plant species belonging to *Aristolochia* and *Asarum* genera are the most reported to contain AA [3,16]. Aristolochic acid I (AAI) and Aristolochic acid II (AAII), two compounds that are structurally different only by the presence of the O-methoxy group at the 8-position for AAI and the absence of this O-methoxy group at the 8-position for AAII (Figure 1) are considered to be the most abundant and active components of AA [17].

The International Agency for Research on Cancer (IARC) classified AA as carcinogenic group I to humans, acting by a genotoxic mechanism [18]. Thus, the use of plants or remedies containing AA has been banned in several countries [19,20,21]. However, due to the worldwide distribution of *Aristolochia* plant species and the great use of herbal medicines in some regions, individuals are still exposed to AA [16,22,23]. In this context, research to understand the molecular mechanisms of AA-induced nephrotoxicity might allow for finding out efficient protective mechanisms or eventually, to provide a model of study for investigating drug-induced organ toxicity. Indeed, since the identification of AA as the causative agent of CHN [11,12], several studies using animal models and cultured cell systems have been conducted. However, the comprehensive cellular and molecular action mechanisms of AA-induced nephrotoxicity have not yet been fully elucidated and AAN treatment is therefore limited.

Several studies have reviewed and summarized the mechanisms reported to be involved in AA pathogenesis, but only few have covered the whole range of protective or potential protective mechanisms against AA-induced nephrotoxicity. In this review, we discuss the molecular mechanisms of action underlying AA-induced nephrotoxicity, while emphasizing the role of enzymatic biotransformation of AA, which result in potentiating its carcinogenic and nephrotoxic effects, as reported in recent studies. Finally, we address the protective strategies and give a summary of agents and their potential protective mechanisms against AA nephrotoxicity in vitro and in vivo, as found in the literature.

## 2. Enzymatic Metabolization of AA Leading to the Formation of DNA Adducts and Induction of *Tp53* Mutations

Studies in rodents have indicated that after oral administration of AA, the drug is absorbed from the gastrointestinal tract into the blood stream and then distributed throughout the body, as evidenced by the presence of DNA adducts detected in several rodent organs, including stomach, intestine, liver, spleen, lung, and kidney [17,19,24]. It has been suggested that AA is metabolized by oxidation (mainly AAI) and reduction pathways [25,26]. The reduction, primarily the nitroreduction of AAI and AAII leads respectively to N-hydroxyaristolactam I and N-hydroxyaritololactam II, which are the major metabolites found in the urine and feces in animal models and humans [3,27,28]. Besides these major metabolites, minor metabolites such as 8-hydroxyaristolochic acid I (AAIa) have also been reported [26]. The oxidation of AA, especially AAI catalyzed by CYP 450 enzymes, leads to the formation 8-hydroxyaristolochic acid (AAIa), a less toxic compound [26].

**Figure 2:**
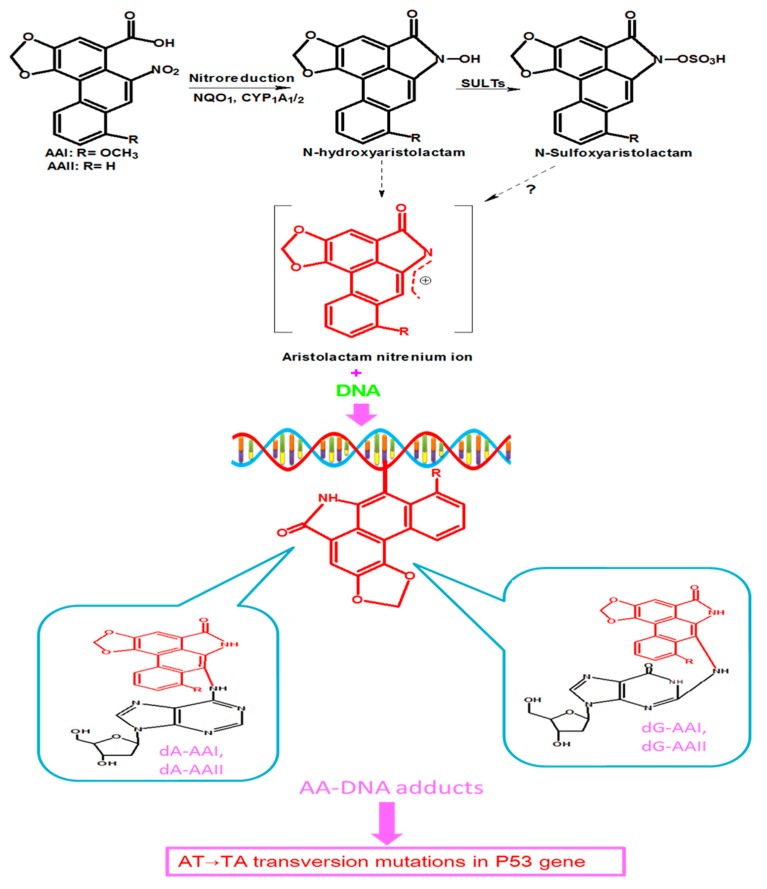
Proposed metabolic pathway of AAI and AAII which leads to their activation. AAI and AAII are reduced by NAD(P)H: quinone oxidoreductase 1 (NQO1), and cytochrome P450 1A1/1A2 (CYP1A1/2) into N-hydroxyaristolactams, which can further bind to DNA to form AA-DNA adducts, including7-(deoxyadenosin-N6-yl) aristolactam I and II (dA-AAI and dA-AAII), and 7-(deoxyguanosin-N2-yl) aristolactam I and II (dG-AAI and d G-AAII). N-hydroxyaristolactams can also react with sulfotransferases (SULTs) to form N-sulfoxyaristolactam, which can be transformed into aristolactam nitrenium ions? (There is still a matter of debate on the conversion of N-sulfoxyaristolactam into aristolactam nitrenium ions, hence the question mark in Figure 2.) These DNA adducts lead to tumor suppressor *Tp53* gene mutations.

Studies on the metabolism of AA have reported the involvement of several enzymes that catalyze the reduction reactions resulting in bioactivation of AA [25,29]. The most important of these enzymes include cytosolic nicotinamide adenine dinucleotide phosphate (NADPH):quinone oxidoreductase 1 (NQO1), microsomal cytochrome P450 (CYP) mainly 1A1, CYP1A2, renal microsome NADPH:CYP oxidoreductase (POR), and prostaglandin H synthase [25,30,31]. The nitroreduction of AAI and AAII leads to corresponding N-hydroxyaristolactams (AL-NOHs) that can be transformed into aristolactam nitrenium ion, a reactive species which reacts with exocyclic amino groups of purine bases of DNA to generate AA DNA-adducts [22], as shown in Figure 2. These adducts include 7-(deoxyadenosin-N6-yl) aristolactam I or II (dA-AAI or dA-AAII) and 7-(deoxyguanosin-N2-yl) aristolactam I or II (dG-AAI or d G-AAII) [22,29,30,32,33]. CYP1A1 and CYP1A2 also catalyze the oxidation of AA, resulting in detoxification products [26,29].

Besides the above-mentioned enzymes, some studies have reported that sulfotransferases are necessary to generate AL-DNA adducts at high levels [34,35,36]. Recently, a study by Chang and colleagues, using microphysiological systems (organs-on-chips), demonstrated that sulfotransferases (SULTs) were involved in enhancement of AA nephrotoxicity. They showed that, after nitroreduction of AAI mediated by NQO1 into aristolactam I (AL-I), the latter was converted into aristolactam-N-sulfate (AL-NOSO3H), a toxic metabolite that was transported out of the liver via multidrug resistance-associated protein (MRP) transporters, and then taken into the kidney via organic anion transporters (OATs), and formed high DNA adducts levels [37].

The role of N-acetyltransferases (NATs) in AA toxicity has also been explored. In this context, Okuno and co-workers recently examined the role of conjugation reactions in the genotoxicity of N-hydroxyaristolactams in *Salmonella typhimurium umu* tester strains expressing human NATs and SULTs and reported that N-hydroxyaristolactams showed stronger genotoxic effects in *umu* strains expressing human NAT1 and NAT2 than in the parent. Strains expressing human SULT1A1 and SULT1A2 also showed increased genotoxicity of N-hydroxyaristolactams [38]. On the contrary, using native enzymes present in human cytosols and human recombinant enzymes including SULTs and NATs, Martinek and colleagues reported that these enzymes of phase II were not involved in AA bioactivation [39]. Additionally, another study reported that in transgenic mice carrying the functional human *SULT1A1–SULT1A2* gene cluster and *Sult1a1* (−/−) mice, sulfoconjugation catalyzed by human SULT1A1 and murine Sult1a1, did not contribute to the activation pathways of AAI and AAII in vivo [40].

The tumor suppressor *Tp53* gene plays a key role in carcinogenesis, especially in human cancers, in which it is found to be mutated up to 50% [41]. Endogenous factors and certain environmental carcinogens have been reported to be the causes of *Tp53* mutations, and AA is one of the environmental mutagens that induce mutations in the *Tp53* gene [42]. As previously mentioned, AA is enzymatically reduced to reactive intermediates that generate DNA adducts, of which dA-AAI constitutes the dominant and most persistent AA-DNA adducts. These adducts can accumulate in target organs such as the renal cortex, serving as biomarkers of prior to long-term exposure to AA or cause *Tp53* mutations (Figure 2) in some tissues, such as the upper urinary track [43]. The most reported mutation is A:T to T:A transversion in the *Tp53* gene [22]. Besides DNA adducts formation, it has been suggested that AA could also bind to RNA, leading to RNA adducts [44], which might also contribute to AA-related toxicity. Therefore, accumulating evidence indicates that enzymatic metabolization of AA increases its mutagenic and carcinogenic properties through DNA adducts’ formation.

## 3. Molecular Mechanisms Involved in AA-Induced Nephrotoxicity

The exposure to AA has been associated with AAN, a kind of rapidly progressive interstitial nephritis characterized by increased serum creatinine, profound anemia, and mild tubular proteinuria associated with histopathologic changes showing hypocellular interstitial infiltrate with severe fibrosis and tubular atrophy [3,6]. AAN is frequently associated with carcinoma, mainly the upper urothelial carcinoma (UUC). 

It was reported that patients with a high intake of AA developed disease which could progress to renal failure after one to seven years, and subjects with a lowest daily cumulative dose of AA intake often maintained relatively normal kidney function for a period of two to eight years of follow-up [5,6]. However, the specific mechanism AAN develops is not yet known despite several studies being conducted. Some molecular mechanisms reported to be involved in AA-induced nephrotoxicity are presented below.

### 3.1. Induction of Oxidative/Nitrosactive Stress and Mitochondrial Dysfunction by AA

Reactive oxygen or nitrogen species (ROS/RNS) are products of a normal cellular metabolism and play vital roles in cell signaling and homeostasis. Overproduction of ROS/RNS or depletion of endogenous antioxidative systems, may lead to oxidative/nitrosative stress [45]. Besides endogenous mechanisms, different xenobiotics can induce oxidative stress resulting in cell damage [46], and AA is one among those xenobiotics. In this regard, a number of studies in vitro and in vivo have demonstrated that treatment of cells or animals with AA increases the amount of ROS/RNS.

In their study, Yu and colleagues showed that AA induced oxidative stress leading DNA damage through activation of the mitogen-activated protein kinase kinase/extracellular signal-regulated kinases 1/2 (MEK/ERK1/2) signaling pathway, and depletion of intracellular glutathione (GSH), resulting in cell toxicity [47]. In addition, Romanov et al. [48] reported that AA was capable of inducing apoptosis and cell arrest in phase G2/M through generation of ROS and activation of mitogen-activated protein kinase (MAP kinase). They observed that MAP kinase could activate p38, leading to apoptosis. A decrease of renal antioxidant capacity in the kidney of C3H/He mice treated with AA was reported in a study by Li and co-workers, in which they noticed the accumulation of methylglyoxal and Nε-(carboxymethyl) lysine, as a result of the involvement of oxidative stress in AAN [49]. Oxidative stress associated with inflammation was also observed in a mouse model of AA-induced acute kidney injury by Declèves and co-workers [50]. Recently, a study has indicated that AAI-induced oxidative stress in C57BL/6N mice was associated with an increase in NADPH oxidase 2 (NOX2) and CYP2E1 expression, and a decrease in catalase, superoxide dismutase, and glutathione synthetase, suggesting that AA might interact with these enzymes to trigger oxidative stress [51]. 

Evidence that AA causes mitochondria damage has been suggested by some experimental studies. In their study, Pozdzick and co-workers demonstrated that AA caused a defective activation of antioxidative enzymes and mitochondrial damage, resulting in tubulotoxicity in the rat model of AAN [13]. Moreover, it was observed that treatment of podocytes with AA was associated with mitochondrial dysfunction and an increase of oxidative stress [52]. AA was reported to reduce the respiratory control ratio (RCR) and adenosine triphosphate (ATP) contents associated with impairment of activity of respiratory complex I in a dose-dependent manner [53]. Treatment of human proximal tubular epithelial (HK-2) cells for 24 h with AAI caused a decrease in cellular ATP, mitochondrial membrane depolarization, cytochrome c release, and an increase of caspase-3 activity [54]. The involvement of oxidative stress in AA-induced cytotoxicity has also been evidenced by various studies, in which antioxidative compounds reducing AA-generated ROS resulted in attenuation of AA-induced cytotoxicity (further section below). Therefore, AA may induce oxidative stress by interacting with NADPH oxidase or antioxidative enzymes (GSH, SOD, catalase).

### 3.2. Induction of Apoptosis

A certain number of studies, conducted both in animal models and in cell culture systems, have indicated that AA induces apoptosis in renal tubular cells. In cell culture systems, AA has been reported to induce apoptosis by suppressing the PI3K/Akt signaling pathway, reducing Bcl-2 levels, and increasing Bcl-2-associated X protein (Bax) levels in human umbilical vein endothelial cells (HUVECs) [55]. Similarly, a study by Xie and colleagues indicated the activation of the PI3K/Akt signaling pathway by relaxin-attenuated apoptosis in HK-2 cells treated with AAI [56]. Recently, an in vivo study showed that relaxin prevented the increase in blood urea nitrogen (BUN) and plasma creatinine, and attenuated renal ultrastructural lesions generated by AAI in C57BL/6 male mice. In vitro, relaxin activated the PI3K/Akt signaling pathway, increased eNOS expression, and inhibited caspases 3 activation, thereby protecting cells against AAI-induced apoptosis in embryonic kidney cells 293 [57]. Additionally, Okada and co-workers reported that treatment of murine tubular epithelial cells (mProx24) with AAI increased the expression of pro-apoptotic protein Bax while decreasing the expression of Bcl-xL, an antiapoptotic protein, resulting in apoptosis induction [58]. A study by Zhou et al. [59] reported that AA induced apoptosis in tubular epithelial cells (TECs) through dephosphorylation of signal transducer and activator of transcription 3 (STAT3) and activation of p53 signaling pathways, and that in *p53*-deficient mice treated with AA, it resulted in massive apoptotic and necrotic TECs death. Zhu and al. [60] observed that exposure of HK-2 cells to AAI increased the oxidative stress, which led to endoplasmic reticulum stress (indicated by increased expression of eukaryotic initiation factor-2*α* (eIF2*α*), glucose-regulated protein (GRP) 78, and CAAT/enhancer-binding protein-homologous protein (CHOP)) and apoptosis.

Pretreatment with N-acetyl-L-cysteine, glutathione or 4-phenylbutyrate (4-PBA), or salubrinal (Sal) significantly inhibited AAI-induced apoptosis, suggesting that ER stress is implicated in AAI-induced apoptosis. Exposure of renal epithelial-like pig kidney cell line (LLC-PK1) to AAI induced an increase in their intracellular calcium concentration leading to endoplasmic reticulum (ER) and mitochondrial stress, which in turn causes activation of the caspase pathway, and finally, apoptosis [61]. The role of intracellular Ca^2+^ in AA-induced apoptosis has also been suggested by other studies [54,62].

Apoptosis was also observed in renal tubular epithelial cells (NRK52E) treated with AA [63], in cultured LLC-PK1 [64], in proximal tubular epithelial cells (PTECs) by Pozdzik and colleagues [13], and in HK-2 cells by Wang et al. [65]. AA has been reported to increase the activity of caspases, resulting in apoptosis generation [4,66]. Yang et al. [67] reported that AAI triggered the mitochondrial/caspase apoptotic pathway in LLC-PK1 cells, which was indicated by an enhanced Bax/Bcl-2 ratio, loss of mitochondrial membrane potential, cytochrome C release, and caspase-3 activation. 

**Figure 3:**
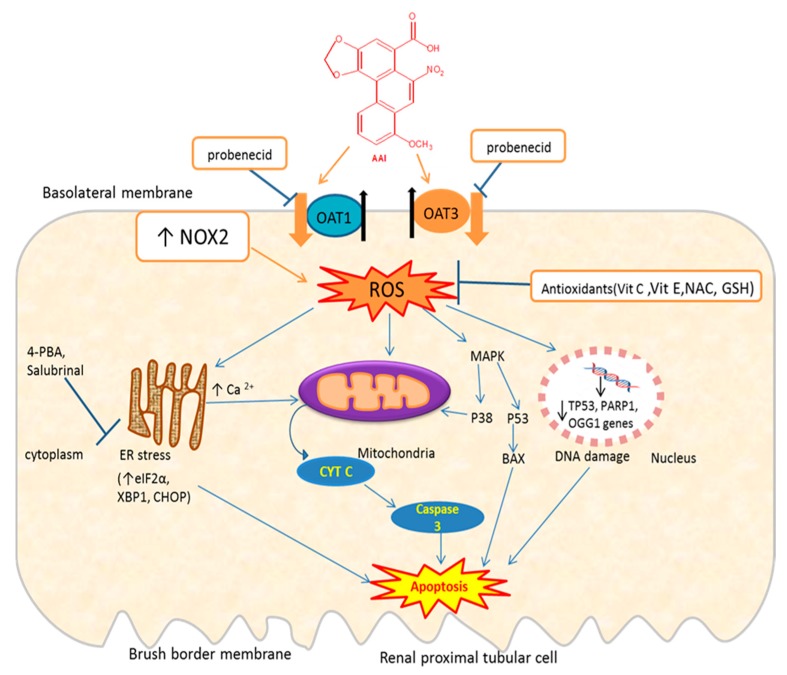
Schematic representation of mechanisms of AAI-induced oxidative stress and apoptosis and protective mechanisms. OAT: organic anion transporter; ROS: reactive oxygen species; NAC: N-acetylcysteine; MAPK: mitogen-activated protein kinase; 4-PBA: 4-phenylbutyrate; Bax: Bcl-2-associated X protein; eIF2*α*: eukaryotic initiation factor-2α; CHOP: CCAAT-enhancer-binding protein homologous protein; XBP1: X-box binding protein 1; *OGG1*: 8-Oxoguanine glycosylase gene; *PARP1*: poly [ADP-ribose] polymerase 1 gene; *Tp53*: tumor suppressor gene; ↑= increase; ↓= decrease; ┴ = inhibition; the thick orange arrows indicate the entry of the toxic compound AAI into the cell; the thick black arrows indicate the exit of endogenous molecules (e.g., glutarate) from the cell. Exposure of proximal tubular cells to AAI, which enter these cells through OAT1/OAT3, increases ROS which can lead to DNA damage (downregulation of DNA repair genes: *Tp53*, *OGG1*), endoplasmic reticulum (ER) stress leading to an increase in Ca^2+^, which in turn affect mitochondria that release Cytochrome C (Cyt C). Cyt C activates caspase-3 leading to apoptosis. AAI-induced ER stress increase some of its protein complex (eIF2a, CHOP, GRP78), which can lead to apoptosis. AAI activates MAPK, which in turn activates p38 or p53 leading to apoptosis. AAI can increase NOX2 activity leading to ROS. Probenecid inhibits the entry of AAI through OAT1/OAT3. Antioxidants (vit E, vit C, NAC, GSH) decrease ROS generated by AAI.

Therefore, there is evidence that AAI, once entering the renal cell through OATs [68,69], trigger oxidative stress which induces apoptosis through induction of ER and mitochondria stress, DNA damage, or activation of the MAPK pathway (Figure 3). AAI may also induce apoptosis through inhibition of the PI3K/Akt signaling pathway.

### 3.3. AA Induces Inflammatory Response 

Several studies have shown that inflammatory mechanisms are strongly linked to the pathogenesis of AA nephrotoxicity. The presence of mononuclear inflammatory cells in chronic renal scarring in rats was reported [14]. In a mouse model of AAN, Honarpisheh and colleagues showed that AA induced severe acute tubular necrosis followed by inflammation and fibrosis. They observed that a persistence of interstitial inflammation during the chronic phase was associated with increased kidney injury and F4/80^+^ macrophages [70]. A similar study demonstrated that progressive peritubular accumulation of monocytes/macrophages and CD8^+^ T cells were the main process linking the transient tubular necrosis phase and tubular atrophy to the tubulointerstitial fibrosis phase [13]. Recently, Baudoux et al. [71] have reported that AAs increase the proportion of myeloid CD11^bhigh^F4/80^mid^ and decrease their counterpart. They showed that CD4^+^ and CD8^+^ T-cells may provide protection against acute tubular necrosis induced by AA. In addition, AA-treated zebrafish embryos showed a blood cell accumulation in the kidney that triggered inflammation, which was evidenced by upregulation of the expression of proinflammatory genes, including tumor necrosis factor alpha (TNFα), cyclooxygenase (COX-2), myeloperoxidase (MPO), and interleukin 1 beta (IL-1β) [72]. Similarly, Jadot and colleagues observed an increase in renal mRNA expression of proinflammatory cytokines, including IL-6, IL-1*β*, and TNF*α*, following the treatment of mice with AA [73]. Recently, NLRP3 inflammasome, a multimeric protein complex that initiates an inflammatory form of cell death, has been reported to be implicated in AA-mediated nephrotoxicity. Studies have shown that AA exposure of Bagg Albino mice (BALB/c mice) and HK-2 cells aberrantly activated NLRP3 inflammasome. Inhibition of IL-1*β* and NLRP3 inflammasome activation by IL-1Ra significantly alleviated renal tubular injury and renal function impairment in AA-induced nephropathy. Moreover, NLRP3 or Caspase-1 deficiency showed protective effects against renal injury in the mouse model of acute AAN, suggesting the involvement of NLRP3 signaling in the pathogenesis of AAN [74]. Inflammation in a rat model of AA was also reported by Kholia et al. [75].

### 3.4. Fibrosis

Fibrosis is a final pathological characteristic of the majority of chronic inflammatory diseases [76]. As reported by several studies, the acute phase of AAN is followed by persistent interstitial inflammation which leads to fibrosis [2,14,70]. Studies that addressed the molecular mechanisms of fibrosis in AA-induced nephrotoxicity have shown that AA can induce fibrosis via activation of TGF-*β*-dependent and c-Jun N-terminal kinase (JNK)/MAP kinase-dependent signaling in mice [77]. Similarly, Li and colleagues observed a significant increase in TGF-*β*_1_ expression associated with interstitial fibrosis in mice treated with AA for 30 days [78]. In addition, it was demonstrated that a blockade of the TGF-*β* signaling pathway ameliorated the impairment of renal function and partially prevented the epithelial–endothelial axis activation and reduction of pericytes-derived PDGFR*β*^+^ perivascular cells accumulation in a rat model of AA-induced acute kidney injury [79]. In their study, Pozdzik and colleagues observed, in mice treated with AA at day 35, the accumulation vimentin, *α*-smooth muscle actin-positive cells, and overexpression of TGF-*β* [13].

Recently, Ye et al. [80] demonstrated that AA induced inflammation and fibrosis through activation of C3a complement systems. Twist1 is a basic helix–loop–helix transcription factor implicated in EMT and plays important roles in various fibrotic diseases [81]. A recent study has reported that in the distal nephron of AA-treated wild-type (WT) mice, Twist1 promoted kidney fibrogenesis by driving renal accumulation of CD64^+^ macrophages and their FN-dependent production of IL-1*β* and monocyte Chemoattractant Protein-1 (MCP-1), and that these effects were reduced in *Twist1* knockout mice, suggesting the role of Twist 1 in AA-induced kidney injury [82].

**Figure 4:**
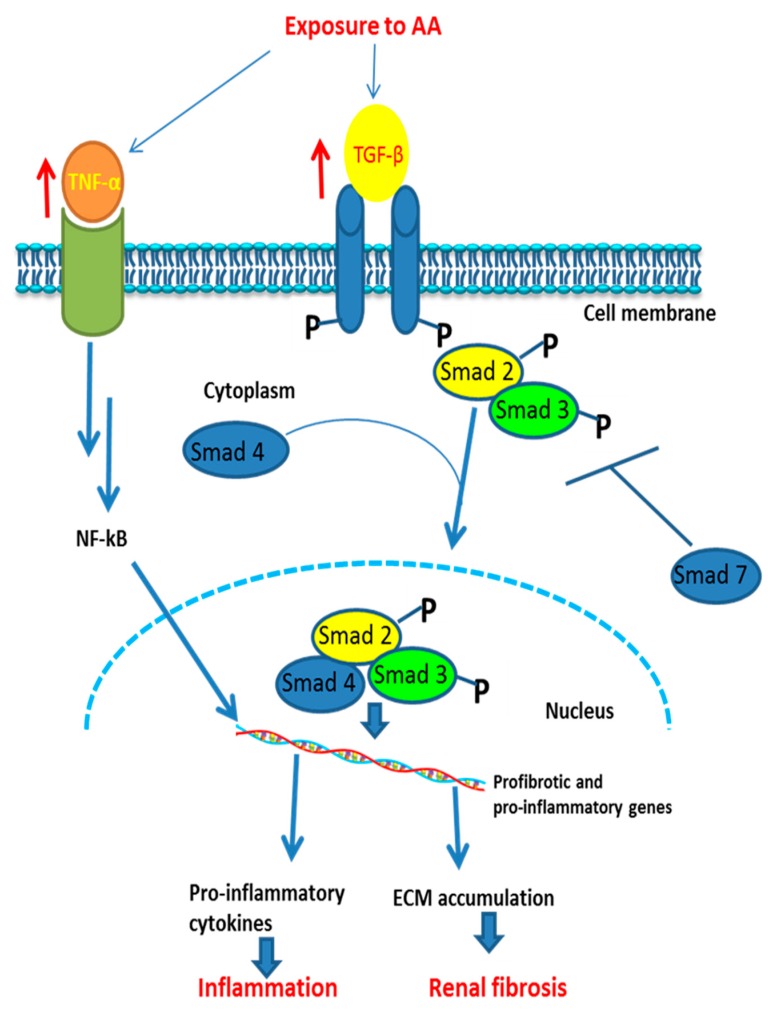
Proposed signaling pathway involved in AA-induced inflammatory responses and fibrosis in an animal model. ↑: increase of activation (of receptors); ┬ = inhibition; NF-κB: nuclear factor kappa-light-chain-enhancer of activated B cells; ECM: extracellular matrix; Smad: mothers against decapentaplegic homolog. Exposure to AA activates tumor necrosis factor (TNF) and transforming growth factor beta (TGF-*β*) which trigger a cascade of reactions leading to activation of proinflammatory and profibrotic genes, then to inflammation and fibrosis, respectively.

Figure 4 shows the signaling pathway of AA-induced inflammation and fibrosis. 

## 4. Approaches and Potential Protective Mechanisms against AA Nephrotoxicity

Since the occurrence of AAN in the 1990s, several studies have been conducted in order to elucidate the cellular and molecular mechanisms of AAN, and possible treatments. However, besides several compounds tested on animals or cultured cell systems, the treatment using steroids, which showed limited therapeutic response consisting of delaying the progression to end-stage renal disease, has been the only therapy tested in humans affected by AAN [6].

Thus, several animal models and cell culture systems reproducing human AAN continue to be established. A number of agents have been tested in order to assess whether they could protect animals or cells against AA’s toxic effects. Most of these animal studies have focused on a preventive approach, consisting of administration of treatment against AAN before or concomitantly inducing AAN. Moreover, most of these studies have investigated the acute phase of exposure to AA but neither the chronic phase nor human experiment.

Since patients with AAN were often diagnosed during routine clinical examination, a curative approach consisting of animal treatment after inducing AAN (onset of AAN) should be taken into account. Among these tested agents, some have shown protective effects against AA-induced toxicity both in vitro and in vivo. Those agents (Table 1) target: (1) metabolism of AA: inhibition or activation of some hepatic enzymes has shown modification of toxic effects of AA; (2) uptake of AA by renal cells: organic anion transporters are reported to be involved in AA uptake into renal tubular cells, and inhibition of these transporters might result in modification of AA effects; (3) oxidative stress: a number of studies conducted in vivo and in vitro have shown the capacity of antioxidants to reduce oxidative stress, thereby reducing the toxic effects of AA; (4) inflammation: anti-inflammatories, namely steroids, have been tested in humans affected by AAN; (5) fibrosis: some compounds have been reported to attenuate fibrosis process in rodent models of AA-induced toxicity; (6) apoptotic pathway, and (7) other signaling pathways.

### 4.1. Agents Interfering with Metabolism of AA

#### 4.1.1. Agents Inducing the Activity of AA-Metabolizing Enzymes

Increasing of expression of enzymes metabolizing AA influences the outcome of its toxicity. Some compounds, including *β*-naphthoflavone, Tanshinone I, 3-methylcholanthrene, baicalin, omeprazole, and Sudan I have been tested to this end.

*β*-Naphthoflavone is an aryl hydrocarbon receptor (AHR) agonist that induces the expression of CYP1A1 [83]. As previously mentioned, CYP1A1 is involved in oxidation of AA, which leads to the products of detoxification of AA. In this regard, Xiao and colleagues showed that treatment of C57BL/6 mice with β-naphthoflavone induced hepatic and renal CYP1A1 or hepatic CYP1A2 expression, leading to an increase in AA metabolization to less toxic metabolites, thereby protecting mice against AA-induced nephrotoxicity [84]. 

Tanshinone I, another inducer of CYP1A1, has been tested on a C57BL/6 mouse model of AAN. Pretreatment with tanshinone I has shown protective effects against AA-induced acute kidney injury, notably through an increase in AA metabolization by CYP1A1 [85].

Additionally, Xue and co-workers demonstrated that treatment of C57BL/6 mice with 60 mg/kg of 3-methylcholanthrene 24 h before exposure to 10 mg and 20 mg/kg of AAI for 3 days or 14 days showed that 3-methylcholanthrene induced an increase in CYP1A1/2 activity, which increased the clearance of AAI, resulting in the reduced AAI toxicity in the kidney [86].

Baicalin is a natural flavonoid extracted from *Scutellaria baicalensis*, and exhibits inducing effects on CYP1A1/2. It has been observed that pretreatment of C57BL/6 mice with baicalin (80 mg or 160 mg/kg) followed AA (10 mg/kg)-attenuated AA nephrotoxicity, notably by increasing the hepatic expression of CYP1A1/2, which in turn led to the increase in metabolization of AA to less toxic metabolites [87].

Omeprazole, which is a proton pump inhibitor with CYP450-inducing effects, particularly on CYP1A1/2, has been reported to alleviate the nephrotoxic effects of *Aristolochia manshuriensis Kom* in rats and mice, by inducing hepatic CYP1A1/2 and renal CYP1A1 expression. CYP1A1/2 is suggested to increase AA metabolization, leading to its elimination. HK-2 cells with ectopic expression of CYP1A1 were also found to be more tolerant to AA than the control cells [88].

Sudan I (1-phenylazo-2-naphthol), known as a potent inducer of CYP1A1/2, has shown protective effects against AA toxicity in rats, through induction of CYP1A1/2 expression. It has been observed that pretreatment of wistar rats with 30 mg/kg of Sudan I induced CYP1A1/2 expression, which led to the increase in AA oxidation, and subsequently, to the decrease in AAI-DNA adducts’ level in rats treated with AA [89].

#### 4.1.2. Agents Inhibiting the Activity of AA-Metabolizing Enzymes

NQO1 is involved in reductive activation of AA, which leads to an increase in its toxicity.

Dicoumarol is known as an NQO1 inhibitor. A study by Chen and his colleagues reported that inhibition of NQO1 (an enzyme known to actively reduce AA) by dicoumarol, suppressed the nitroreduction of AAI and the formation of aristolactam I (which in turn can be converted into toxic metabolite) in renal tissue. The inhibition of this pathway by dicoumarol or phenindione (another inhibitor of NQO1) protected mice against AA nephrotoxicity [90].

Another study, by Chang and colleagues, reported that G lycine N-methyltransferase (GNMT) exerted protective effects against AA-induced nephropathy in female mouse hepatocytes by reducing NQO1 expression and increasing CYP3A44 expression [91].

### 4.2. Agents Exerting Antioxidative Effects

Tocopherol (vitamin E) is a natural antioxidant compound. A study by Wu and his colleagues demonstrated that, by reducing oxidative stress and the caspase-3-dependent apoptosis pathway in cultured NRK-52E cells treated with AA, tocopherol attenuated AA-induced toxicity [64].

Vitamin C is another natural antioxidant compound. It has been reported that pretreatment with vitamin C exerted protective effects on NRK-52E treated with AA, through decreasing the high ratio of hydrogen peroxide (H_2_O_2_) and caspase-3 activity generated by AA [92]. 

Liver-type fatty acid binding protein (L-FABP) is a protein with endogenous antioxidative function [93]. It has been reported that human liver-type fatty acid binding protein (hL-FABP) transgenic (Tg) mice treated with AAI showed a lower level of urinary Nε-(hexanoyl) lysine (considered as a lipo-oxidative stress marker during the initial stage of oxidative stress), production of heme oxygenase-1, and ameliorated renal dysfunction compared with wild-type (WT) mice treated in the same conditions, suggesting that L-FABP protected mice against AA-induced nephrotoxicity by decreasing oxidative stress [93].

### 4.3. Agents Inhibiting Renal Uptake of AA

Probenecid is a drug used in the treatment of gout and hyperuricemia. It blocks the entry of organic anion transporters and then prevents the uptake of compounds into the cells [94]. In this context, Baudoux and co-workers observed that in vitro, using probenecid inhibited AAI entry through organic anion transporters, reduced specific AA-DNA adducts’ formation, and preserved cellular viability. They observed that in a mouse model of AAN, treatment with probenecid prevented the increase of plasma creatinine and tubulointerstitial injuries, and also attenuated the severity of ultrastructural lesions caused by AA [95].

### 4.4. Agents Targeting AA-Induced Inflammatory Response

Steroids have been the only compounds with anti-inflammatory properties to be tested in human therapy. Prednisolone has been used and has shown its efficacy by delaying the progression to end-stage renal disease [6,96].

Resveratrol and ursolic acid are natural compounds exhibiting antioxidative and anti-inflammatory properties [97]. In their study, Ding and colleagues reported that resveratrol and ursolic acid attenuated AA-induced nephrotoxicity in zebrafish by preventing blood cell accumulation in the kidney, improving the glomerular filtration rate and suppressing the expression of pro-inflammatory genes *TNFα* and *MPO* [98].

### 4.5. Agents Attenuating Fibrosis

Mothers against decapentaplegic homolog 7 (SMAD7) has been reported to attenuate the development of fibrosis by inhibiting TGF-*β* signaling [99]. In their mouse model of AAN, Dai and co-workers showed that the loss of SMAD7 was associated with activation of TGF-*β*/Smad3 and NF-κB signaling pathways, resulting in aggravation of progressive fibrosis and inflammation in mice treated with AA, and that restoration of SMAD7 locally in the kidneys of Smad7 knockout mice inactivated TGF-*β*/Smad3-mediated renal fibrosis and NF-κB-driven renal inflammation, thereby preventing the progression of chronic AAN [100]. 

Similarly, another study reported that inhibition of TGF-*β* by neutralizing anti-TGF-*β* antibody (1D11) ameliorated the impairment of renal function in AA-induced acute kidney injury in rats [79]. 

Selective tyrosine kinase inhibitor of macrophage-colony stimulating factor (fms-I) activity of the macrophage colony-stimulating factor (c-fmc) receptor was reported to decrease the progressive interstitial fibrosis and renal inflammation in AA-treated mice, notably by inhibition of TGF-*β*/Smad3 and NF-kB signaling pathways [101]. 

Hepatocyte growth factor (HGF) has been reported to exert an inhibitory effect on TGF-*β*, resulting in prevention of initiation and progression of chronic renal fibrosis [102]. In this regard, Okada and his colleagues showed that transgenic-derived HGF was able to attenuate fibrosis induced by AA in mice [58]. 

Bortezomib is proteasome inhibitor used in the treatment of multiple myeloma. It has been demonstrated that bortezomib exhibits an antifibrotic effect via inhibition of TGF-*β*_1_. A study by Zeniya and colleagues showed that inhibition of TGF-*β*_1_/Smad-3 signaling and apoptosis by bortezomib attenuated AA-induced fibrosis in mice [103].

### 4.6. Agents with Antiapoptotic Effects in AA-Induced Nephrotoxicity

It has been shown that Estradiol can inhibit apoptosis and exerts a renoprotective effect on renal injury in animal experiments [104]. In this regard, Shi et al. [105] observed that 17*β*-Estradiol inhibited apoptosis in HK-2 and mice renal tubular epithelial cells through a decrease of phosphor-p53, p53, and caspace-3 expression. Their study in vivo showed that male mice treated with AA exhibited more severe acute kidney injury than female mice and that the treatment with 17*β*-Estradiol attenuated renal tubular injury and lower serum creatinine both in male and female mice.

Erythropoietin (EPO) is a hematopoietic factor produced mainly by the adult kidney that plays a role in the production of red blood cells. It has been reported that EPO exerts antiapoptotic and tissue-protective effects [106]. In this regard, Wang et al. [107] showed that recombinant human erythropoietin was able to inhibit the activation of caspase-3 and to upregulate the expression of anti-apoptotic gene Bcl-XL.

Bone morphogenetic protein-7(BMP-7) is a member of the TGF-*β* superfamily that possesses antiapoptotic and antifibrobrotic activities. A study in vitro investigating the effects of BMP-7 on cultured HK-2 cells treated with AA showed that BMP-7 exerted protective effects against AA-induced cytotoxicity by inhibition of caspase-3 activation, resulting in a decrease of cell apoptotic rate. BMP-7 also reduced ECM formation through inhibition of profibrotic effects of TGF-*β* [108].

### 4.7. Other Agents

Prostaglandin E1 (PGE1) is a natural prostaglandin with vasodilator and smooth muscle relaxing effects. Nakagawa et al. [109] showed that PGE, together with EP4, could inhibit tubulointerstitial fibrosis. In a Sprague–Dawley rat model of AAN, treatment with PGE1 increased vascular endothelial growth factor (VEGF) expression and microvascular density. PGE1 also prevented capillary loss and relieved hypoxia, resulting in kidney protection against AA-induced nephrotoxicity [110]. 

Bardoloxone methyl (BARD) is a synthetic triterpenoid that activates the nuclear factor erythroid 2-related factor 2 (Nrf2) pathway (which mediates antioxidant and anti-inflammatory response) and inhibits the NF-kB pathway. A study by Wu et al. [111] showed that treatment of C57BL/6 mice with 10 mg/kg/day of BARD by intraperitoneal (I.P) injection increased the expression of renal genes including *Nrf2, PPARγ*, and *HO-1* (genes described to protect against ischemic acute kidney injury) and downregulated Keap1 expression compared with those in the AA-treated group, thereby protecting mice against renal damage induced by AA.

Nitric oxide (NO) is involved in the regulation of renal blood flow. Studies have reported that AAN is associated with decreased NO bioavailability [73]. In this regard, Declèves and colleagues showed that administration of L-arginine, a precursor of NO, restored its bioavailability and ameliorated AKI in mice treated with AA [50]. Additionally, it was reported that L-arginine supplementation also delayed the progression of AKI to CKD in C57BL/6J mice treated with AA [73].

Aquaporin-1 is a membrane water channel that allows rapid water movement driven by a transmembrane osmotic gradient. Inhibition of aquaporin-1 expression by ribonucleic acid interference (RNAi) was reported to protect cells against AAI-induced apoptosis in HK-2 cells [112].

Cyclic helical B-peptide (CHBP) is a derivative of erythropoietin with tissue-protective effects in various organ-induced injuries, but without erythropoietic effects. Treatment of mice with CHBP has shown amelioration of renal tubular injury and inflammatory infiltration, and a reduction of blood urea nitrogen and serum creatinine in a mouse model of AAN. In addition, CHBP exerted inhibition of caspase-3 and 9 and improved the level of the expression of Bcl-xL (a protein encoded by *BCL2-like 1* gene) in vivo [113].

Interleukin-22 is a member of a group of the cytokines IL-10 family which plays a potent role as a mediator of inflammation. It has been recently observed that treatment of mice or cells with AA resulted in activation of NLRP3 inflammasome, which increased renal tubular injury and function loss, and that administration of Interleukin-22, which inhibited activation of NLRP3 inflammasome, ameliorated renal tubular injury [74].

Melatonin is a hormone released by the pineal gland and regulates the sleep–wake cycle. It exerts antioxidant, anti-inflammatory, and antiapoptotic effects [114]. Recently, it has been demonstrated in a mouse model of AAN that treatment with melatonin protected mice against AA nephrotoxicity. It was observed that melatonin decreased oxidative stress (by decreasing the level of malondialdehyde and the expression of NOX2 and CYP2E1), apoptosis (by suppressing the increase in p53, bax), and inflammation (evidenced by decreased expression of TNF-α and IL-6, MCP-1) generated by AAI [51].

Captopril and chymostatin are an angiotensin converting enzyme (ACE) inhibitor and chymase inhibitor, respectively. An in vivo study, using C57BL/6 mice, showed that treatment of mice with AAI (15 mg/kg per day for 3 days, i.p) induced an increase of BUN, serum creatinine, infiltrating inflammatory cells, and tubular atrophy. An increase in chymase activity along with a high level of angiotensin II, but slightly decreased activity of ACE, has been observed. The treatment with 10 mg/kg for 3 days of chymostatin alone, or chymostatin (10 mg/kg for 3 days) with captopril (10 mg/kg for 3 days), reversed these changes and alleviated the progression of nephropathy induced by AAI, suggesting that the renin-angiotensin system is implicated in AAI-induced nephrotoxicity [115].

## 5. Conclusions and Future Perspectives

Herbal drugs containing aristolochic acids (AA) have been used for centuries for medicinal purposes. Due to the nephrotoxic and carcinogenic effects of AA, the use of all products containing AA has been prohibited in many countries. However, the worldwide distribution of *Aristolochia* species, the great utilization of medicinal plants, and the complexity of some traditional pharmacopeia suggest that AA-containing botanicals are still in use in some regions across the world. Despite several studies performed in vitro and in vivo, the mechanisms of AA-induced nephrotoxicity are not yet fully understood, and therapeutic measures are limited. Studies have demonstrated that apoptosis, oxidative stress, and inflammation are among the most implicated mechanisms in AA-nephrotoxicity, and the enzymatic bioactivation of AA is the prerequisite step for its carcinogenic effects.

Several compounds investigated have shown protective effects against AA-induced nephrotoxicity. However, most of those compounds have been tested on the acute phase but not on the chronic phase of exposure to AA or in affected humans. In addition, only preventive approaches consisting of treatment of animals or cells with those compounds before (but not after) inducing AAN have been used. Since AAN cases were often diagnosed during routine clinical examination, a curative approach consisting of animal treatments after inducing of AAN (onset of AAN) and chronic exposure evaluation should be taken into account in the future studies.

Moreover, the agents reported to exert nephroprotective effects against AA-induced nephrotoxicity act at different levels and by different mechanisms of action. The analysis of these nephroprotective mechanisms suggests that: (i) hepatic enzymes metabolizing AA may play an important role in the occurrence of AA nephrotoxicity (the level of enzymes that metabolize AA in an individual may promote or impede the occurrence of AAN after exposure to AA), and (ii) the transport mechanism of AA into the renal cell may greatly contribute to AA-mediated nephrotoxicity.

Thus, further studies combining two or more agents acting at different levels (e.g., inhibitor of AA uptake + enzymatic inducer of AA metabolization) would be a good approach to increase their nephroprotective effects. Some tested agents are recognized as toxic (e.g., 3-methylcholantrene, Sudan I) and should not be considered for possible therapeutic applications. Other agents need further investigation on their toxicological profile and efficacy, because they might serve as potential for pharmacological applications.

Therefore, the complete understanding of the molecular mechanisms of AA-induced nephrotoxicity is of a major importance because it might allow for finding out efficient protective mechanisms against AA nephrotoxicity or eventually provide a study model for investigating drug-induced kidney toxicity.

## Figures and Tables

**Table 1:** Summary of main agents and their protective mechanisms against AA nephrotoxicity.

No	Mechanisms of Action	Agents	Dose, Route of Administration, and Duration of Treatment	Experimental Model	Reference
1	Induction of CYP1A1/CYP1A2 expression	3-methylcholanthrene	60 mg/kg/day, i.p; 3 and 14 days	C57BL/6 mice	[86]
		*β*-Naphthoflavone	80 mg/kg/day; i.p; 3 days	C57BL/6 mice	[84]
		Baicalin	80 mg/kg/day; i.p; 3 days	C57BL/6 mice	[87]
		Tanshinone I	30; 60 mg/kg/day; 3 days	C57BL/6	[85]
		Omeprazole	0.2 mg/kg/day; po; 14 days	ICR mice	[88]
		Omeprazole	2.2 mg/kg/day; 7 days	Sprague–Dawley rat	[88]
		Sudan-I	30 mg/kg/day; i.p	Wistar rats	[89]
	Inhibition of NQO1 expression	Dicoumarol	15 and 30 mg/kg; 2x/day or 60 mg/kg/day	C57BL/6 mice	[90]
2	Inhibition of AA uptake	Probenecid	150 mg/kg body weight, 2x/day; i.p; 2, 4, 5, 8 days	C57BL/6 mice	[95]
3	Inhibition of ROS	Vitamin C	5 µM	NRK-52E cells	[92]
		Vitamin E	10 and 20 µM	NRK-52E cells	[64]
4	Anti-inflammation	Prednisolone	0.75 mg/kg/day; 1 month, then 0.1 mg/kg/day	Humans	[96]
5	Fibrosis attenuation	Bortezomid	0.5 mg/kg/, i.p; 2x/week, 10 weeks	C57BL/6J mice	[103]
		HGF	N/A	HGF tg mouse	[58]
		HGF	10 and 100 ng/mL	mProx24 cells	[58]
		Neutralizing anti- TGF-*β*1 antibody	5 mg/kg/day; 4 days	Wistar rat	[79]
6	Apoptosis attenuation	17-*β* Estradiol	2 mg/kg/day; i.p; 4 days	C57BL/6 mice	[105]
		17-*β* Estradiol	10 and 20 U/mL; 24h	LLC-PK1 cells	[105]
		Erythropoietin (EPO)	500 and 1000 ng/mL; 48h	HK-2 cells	[107]
		BMP-7	0.2 mg/kg /day; 6 days;	C57BL/6 mice	[108]
		Relaxin	100 ng/mL	HK-2 cells	[56]
		Relaxin	100 ng/mL	Kidney cells 293	[57]
7	Nitric oxide modulation	L-arginine	300 mg/mouse/day; 4 days	C57BL/6J	[50,73]

ip: intraperitoneal; po: per os; CYP1A1/CYP1A2: Cytochrome P450 1A1/1A2; NQO1: NAD(P)H:quinone oxidoreductase 1; AA: aristolochic acid; ROS: reactive oxygen species; N/A: not available; HGF: hepatocyte growth factor; TGF-*β*1: transforming growth factor beta 1; BMP-7: bone morphogenetic protein 7; ICR mice: albino mice from Institute of Cancer Research (USA).
